# Do the Brazilian sardine commercial landings respond to local ocean circulation?

**DOI:** 10.1371/journal.pone.0176808

**Published:** 2017-05-10

**Authors:** Mainara B. Gouveia, Douglas F. M. Gherardi, Carlos A. D. Lentini, Daniela F. Dias, Paula C. Campos

**Affiliations:** 1 National Institute for Space Research (INPE), Remote Sensing Division, São José dos Campos, São Paulo, Brazil; 2 Instituto de Física – Departamento de Física da Terra e do Meio Ambiente, Universidade Federal da Bahia - UFBA, Salvador, Bahia, Brazil; 3 GOAT - Grupo de Oceanografia Tropical, Universidade Federal da Bahia - UFBA, Salvador, Bahia, Brazil; Fisheries and Oceans Canada, CANADA

## Abstract

It has been reported that sea surface temperature (SST) anomalies, flow intensity and mesoscale ocean processes, all affect sardine production, both in eastern and western boundary current systems. Here we tested the hypothesis whether extreme high and low commercial landings of the Brazilian sardine fisheries in the South Brazil Bight (SBB) are sensitive to different oceanic conditions. An ocean model (ROMS) and an individual based model (Ichthyop) were used to assess the relationship between oceanic conditions during the spawning season and commercial landings of the Brazilian sardine one year later. Model output was compared with remote sensing and analysis data showing good consistency. Simulations indicate that mortality of eggs and larvae by low temperature prior to maximum and minimum landings are significantly higher than mortality caused by offshore advection. However, when periods of maximum and minimum sardine landings are compared with respect to these causes of mortality no significant differences were detected. Results indicate that mortality caused by prevailing oceanic conditions at early life stages alone can not be invoked to explain the observed extreme commercial landings of the Brazilian sardine. Likely influencing factors include starvation and predation interacting with the strategy of spawning “at the right place and at the right time”.

## Introduction

In eastern boundary current systems (EBC), interdecadal variability of sardine populations can be related to both sea surface temperature (SST) anomalies and flow intensity [1]. During periods of weaker flow, the residence time of larvae in the EBC and reproduction success increase, whereas that during periods of stronger flow, larval survival is restricted to coastal waters and productivity is relatively low. In addition, mesoscale ocean processes, such as current meandering, eddies and thermal fronts can potentially influence sardine production. However, biological, behavioral and multispecies interactions also play an important role in population variability through mechanisms like undetermined fecundity, temperature preferences, density-dependent processes, trophic dynamics and learning from previous stressful experiences or one trial learning [2].

[3] suggested that in western boundary current systems, such as the Brazil Current (BC) in the South Brazil Bight (SBB), the reproductive strategy of the Brazilian sardine was linked to a stable and enriched environment with a high likelihood of larvae retention. An improved analysis carried out by [4] found evidences that negative SST anomalies can result from the blocking effect of clouds on shortwave solar irradiance during the reproductive season prior to catch maxima. According to [5], the spawning habitat of the Brazilian sardine expands and contracts along the SBB following the interannual variability of the horizontal and vertical mesoscale structure of the ocean. Survival of the Brazilian sardine seems, therefore, to be strongly controlled by wind-forced surface water transport and vertical mixing and respond to specific South Atlantic Convergence Zone (SACZ) configurations.

The ecological relevance of the spawning sites within the SBB suggested by [5] for the Brazilian sardine was tested by [6] against a random spawning strategy. Results showed that the influence of water temperature on larval survival between these two spawning strategies does not differ significantly. However, mortality caused by advection to unsuitable habitats is significantly higher for random spawning. Water temperature is a limiting factor for egg and larval survival and influence interannual variability of the Brazilian sardine spawning process. In particular, the upwelling at the northern portion of the spawning habitat can induce sardine to spawn in the central and southern portions of the SBB [7], not only because of the increase in larva mortality by low temperature, but also because of the offshore Ekman transport associated with the wind-induced upwelling [4].

The hypothesis that remains to be tested is whether it is possible to clearly determine the optimum oceanic conditions that would favour high commercial landings of the Brazilian sardine within the SBB. Or, alternatively, if extremely low landings could be result of specific unfavourable conditions. Fluctuations in fish abundance can be explained by migration, year-class success, fishing and stock-recruitment relationships and adult trophic interactions [8]. In a recent paper, [9] showed that the Pacific Decadal Oscillation (PDO) explains the succession of sardine recruitment in the California Current (an EBC) during the last three decades. Favourable oceanographic conditions for parents both during the feeding season and the spawning season would lead to high recruitment (and sardine abundance). A contrasting view comes from genetic studies suggesting that few individuals contribute to the reproductive success because these would have been spawned “at the right place and at the right time” [2]. This could explain the observed large swings in the total sardine landings as a result of an opportunistic reproductive strategy based on the continual testing for favorable environmental conditions over a large area.

In this paper, we use an individual based modelling (IBM) approach to look for differences in eggs and larvae mortality resulting from oceanic conditions prior to extreme Brazilian sardine landings. This investigation fills an important gap in our understanding of the Brazilian sardine ecology not addressed by previous research [4, 6]. We first describe the datasets and the modelling approach. Ocean model simulations are compared with satellite data and the oceanographic regime of the SBB is briefly discussed. Finally, we test egg and larval mortality due to low water temperature and advection prior to extreme (high and low) sardine landings. Finally, a synthesis is presented with our main findings on the environmental influence on the Brazilian sardine population.

## Materials and methods

The relationship between oceanic conditions during the spawning season and commercial landings of the Brazilian sardine was investigated using a two-step approach. Firstly, we run the Regional Ocean Modeling System (ROMS) to simulate the physical processes in the SBB and, secondly, we used the IBM Ichthyop to simulate ichthyoplankton spawning pattern, dispersion and survival one year before landing. Similar simulation approaches using ROMS and Ichthyop were applied to the identification of spawning patterns of *Sardinella aurita* in the Senegalese-Mauritanian region [10] and ichthyoplankton transport in the African coast [11]. A more ambitious effort was recently proposed by [12], who demonstrated an end-to-end simulation scheme for anchovy and sardine population dynamics using ROMS for the California Current system.

The sardine landing data used here was the total annual biomass landings (in metric tons) recorded between 1980 and 2007 in the landing ports of Santos (SP), Rio de Janeiro (RJ) and Itajaí (SC), where fisheries are concentrated (http://www.fao.org/fishery/species/2090). [13] have argued that this data is representative of the interannual variability of the sardine stock because fishing boats generally lack refrigeration systems and need to rely on ports located close to the fishing areas. Also, sardine stocks tend to concentrate in depths shallower than 60 m, as indicated by acoustic surveys, so that this fisheries heavily rely on close landing harbours.

### Model configuration

The configuration of both the hydrodynamic and the lagrangian (IBM) models are basically the same as used by [6], who compared the simulated spawning and dispersal of the Brazilian sardine eggs and larvae to field data. Therefore, by using the same model scheme, it is possible to assume that the interannual variability of larval mortality presented here has been indirectly validated. The ROMS configuration used to simulate the hydrodynamic of the SBB has a grid domain bounded by 20°S to 30°S and 40°W to 50°W, a horizontal resolution of 1/12° (approximately 9.2 km) and a vertical discretization of 30 sigma levels. Model forcing used shortwave radiation surface fluxes, precipitation, atmospheric pressure, relative humidity, air surface temperature and wind velocity at 10 m, from the Climate Forecast System Reanalysis (CFSR) dataset [14]. The ocean lateral boundaries are open in the east and south and were forced using temperature, salinity, current velocities and sea surface height monthly means obtained from Simple Ocean Data Assimilation (SODA) [15]. This ROMS configuration has shown to reproduce the main of mesoscale phenomena in the SBB, including eddies and thermal fronts [6]. A spin up experiment was run for five years between January 1980 and December 1985 and the last month was used to run a 27 years long experiment (1980–2007).

**Fig 1:**
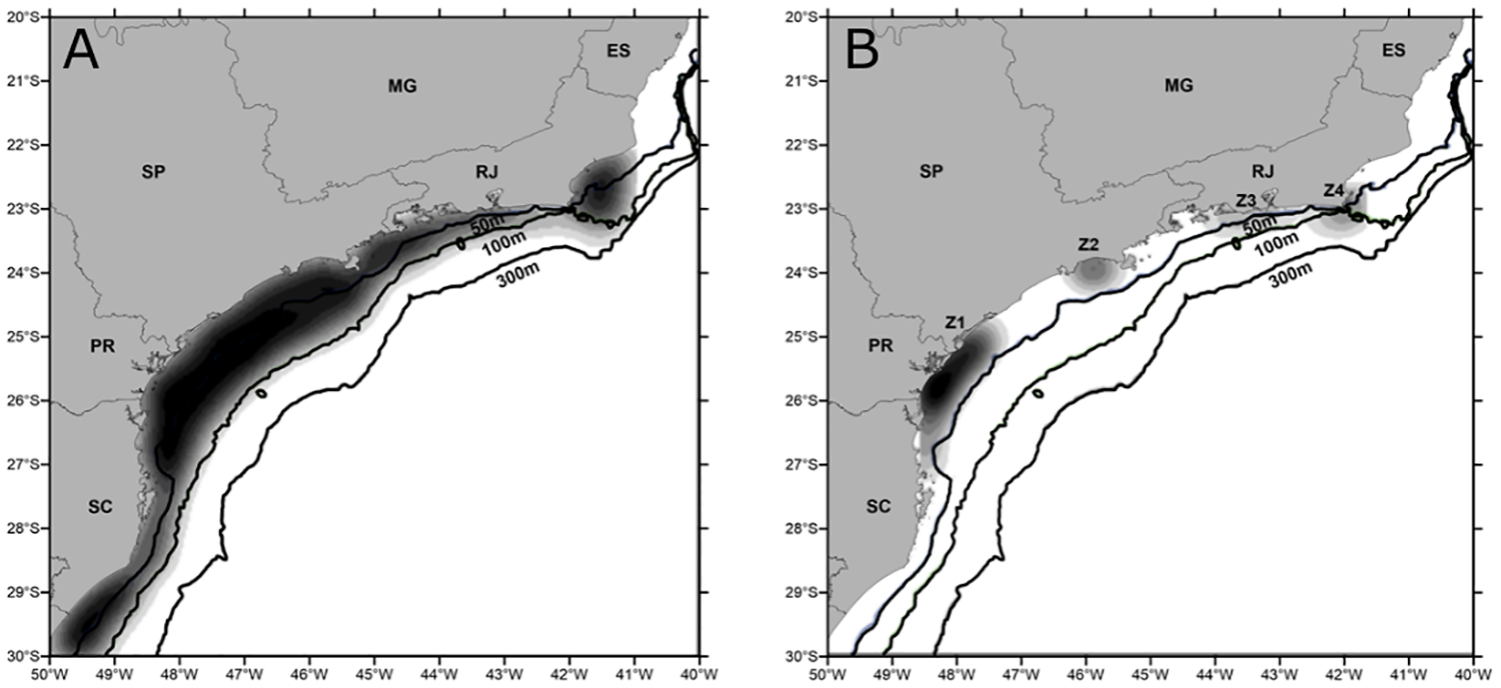
Spawning IBM. Spawning scenarios: left map shows random spawning and right map shows the locations (Z1 to Z4) used as egg release zones. The grey levels indicate the kernel density of the eggs released in each experiment. Numbers in depth contour lines are in meters.

We conceived IBM experiments to test if eggs and larvae mortality could have been influenced the total annual landings of sardine in two different spawning scenarios: one based on four pre-defined release zones and another where eggs were released randomly along the SBB (Fig 1). Each experiment was initiated with the release of 30,000 eggs in surface waters shallower than 100 m. This is intended to offer a testable representation of egg and larvae mortality rates between contrasting spawning strategies. Individuals were tracked in space and time for 45 days, after which larvae are considered as pre-juveniles [16]. Larvae are not expected to be active swimmers during the experiments, showing only diel vertical migration. The biological parameters considered in the experiments are (detailed below): (1) advection and diffusive horizontal movements; (2) vertical movement for eggs due to buoyancy; (3) larval diel vertical migration (DVM); (4) mortality by advection when larvae reach areas with depths greater than 200 m; and (5) mortality by lethal temperature.

The horizontal advection formulation uses a Forward Euler scheme that considers both zonal and meridional velocity components, following Eqs (1) and (2):

$$xp_{t+\Delta t}=xp_{t}+u\Delta t$$ (1)

$$yp_{t+\Delta t}=yp+v\Delta t$$ (2)

where *xp*, *yp* define the horizontal position of the particle (m); *u*, *v* are the zonal and meridional components of currents velocities (m s^-1^); and *Δt* is the time step (s). The diffusive horizontal movement is governed by Eqs (3) and (4), based on the lagrangian coefficient of horizontal diffusivity, which is a function of the turbulent dissipation rate and the grid cell size (Eq (5)), and a random factor, obtained from the Marsenne Twister random number generator, responsible for introducing a certain randomness to the trajectories [17]. We used a turbulent dissipation rate *ε* of 7.4 x 10^−8^ m^2^ s^-3^, which is representative of an environment located at 20 m depth, under a 6 to 7 m s^-1^ wind velocity [18, 19]:

$$dx=Rx\sqrt{2\Delta tK_{h}}$$ (3)

$$dy=Ry\sqrt{2\Delta K_{h}}$$ (4)

$$K_{h}=\varepsilon^{\frac{1}{3}}l^{\frac{4}{3}}$$ (5)

where *dx*, *dy* are the zonal and meridional horizontal displacement of a particle; *Rx*, *Ry* are random numbers generated by the Marsenne Twister method, ranging between [-1;1]; *K*_*h*_ is the lagrangian coefficient of horizontal diffusion (m^3^ s^-3^); *l* is the grid cell size (m); *ε* is the turbulent dissipation rate (m^2^ s^-3^). We used 10^−9^ m^2^ s^-3^, as suggested by [17].

Egg buoyancy is calculated based on the vertical velocity for prolate spheroids, which is a function of the gravitational force, sea water and egg densities, water molecular viscosity and the minor and major axes of prolate spheroids [20]. The vertical movement of larvae initiates two days after hatching, when the larvae gains motility [16]. During the night larvae are positioned at 5 m depth and at 30 m during the rest of the day [21]. This instantaneous depth change correponds to a vertical speed of under 7 mm s^-1^.

Our simulations considered two sources of mortality: (1) lethal water temperature, when larvae are exposed to water temperatures <17°C (for eggs) and <16.5°C (for larvae) [22]; and (2) loss by advection, if larvae are located in areas deeper than 200 m or out of the model domain by the end of simulations. Beyond this depth geographical limit larvae tend to die due to starvation [23]. Larval survival rate was calculated relative to the total number of individuals released excluding the individuals that died by temperature or were advected offshore.

**Fig 2:**
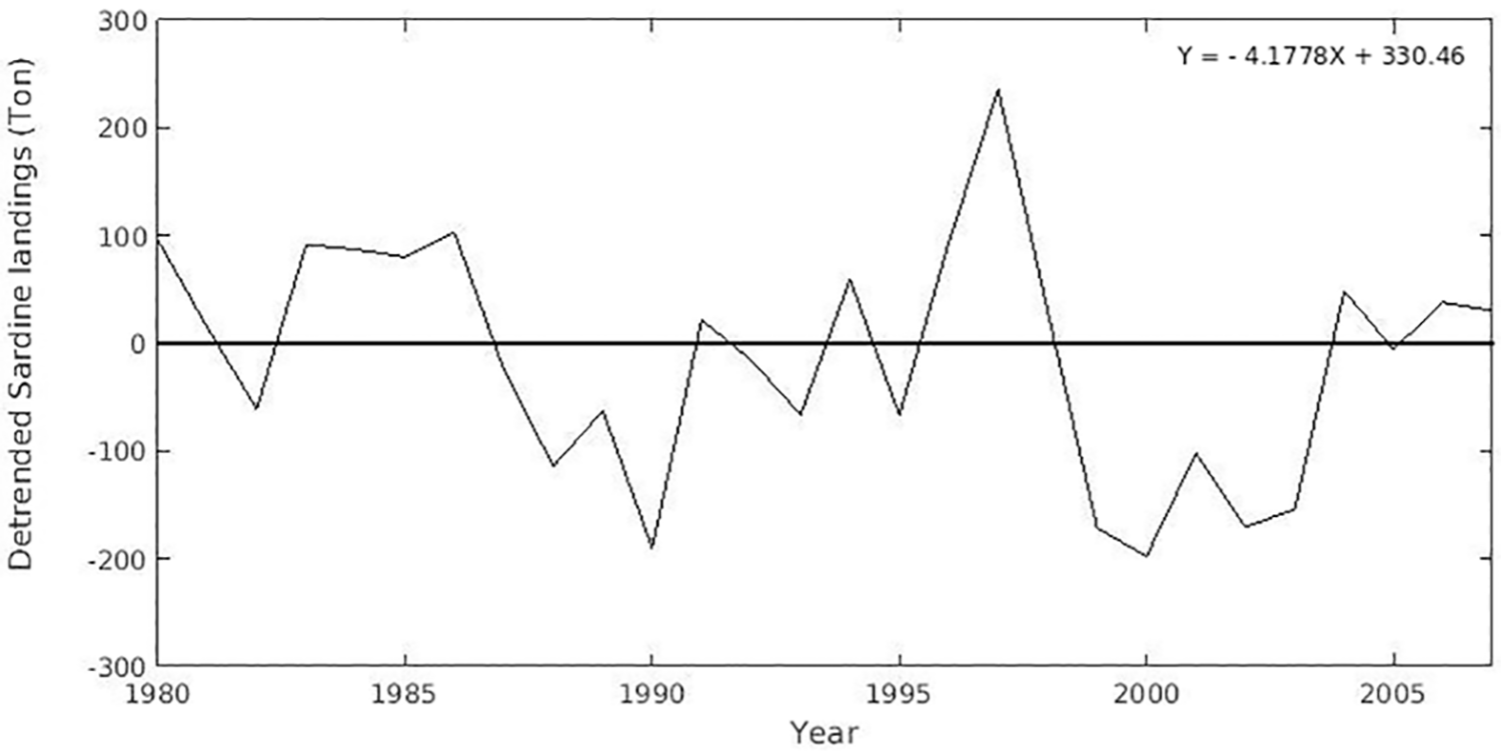
Fish landings. Detrended time series of the total annual landings of the Brazilian sardine.

For the pre-defined spawning zones, the total amount of eggs released (30,000) was distributed proportionally to the size of each zone (the larger the size, the more eggs are released). These spawning zones were defined based on spatial models of egg density that generated a probability map of egg occurrence [5]. Particles were released at sites with probability of finding sardine eggs higher than 0.75, although these can be found over most of the continental shelf, limited by the 200 m isobath. Experiments were conducted separately for years of maximum and minimum landings (Fig 2), which were selected based on the estimation of the generalized extreme value (GEV) parameters [24, 25]. The Weibull distribution was chosen based on the GEV shape parameter and successfully tested against the Weibull distribution using the one-tailed Kolmogorov—Smirnov statistic test (α = 0.05, n = 27), with D_obs_ = 0.25 and critical D = 0.79 [26]. The years of minimum landings were: 1988, 1989, 1990, 1993, 1995, 1999–2003, 2005 e 2007 and maximum landings occurred in 1983–1986, 1996 and 1997.

The differences in mortality rates, between the reproductive periods prior to maximum/minimum landings due to lethal temperature and loss by advection, were tested using the non-parametric Kruskall-Wallis test [26]. Spatial structuring may be favoured by spawning within specific zones, so we also tested for differences between random and zone spawning with respect to mortality rates by low lethal temperature and advection.

### Model accuracy

Model accuracy was evaluated comparing horizontal maps of seasonal SSTs (i.e., austral summer—JFM—and winter—JAS) with AVHRR data (Pathfinder Program, version 5.0, Level 3) from 1980 to 2007. Monthly satellite-derived SST maps have a horizontal resolution of 4 km and a full description of the Pathfinder dataset can be found on the PO.DAAC website at http://podaac.jpl.nasa.gov/AVHRR-Pathfinder. Monthly images were used to generate the seasonal mean data for both the horizontal maps and the longitudinal profiles. For comparison purposes, model SST outputs were regridded to fit the Pathfinder data lower spatial resolution. Two latitudinal profiles of SST, one north of Cabo Frio (22°S) and another south of Cabo de Santa Marta (29°S) were also used to compare model and satellite data.

The model's seasonal surface velocity output was compared with OSCAR data to assess its accuracy. The OSCAR (Ocean Surface Current Analyses Real-time) program produces satellite-derived ocean surface velocity fields averaged over the top 30-m of the ocean's surface. This data is provided on a global grid every ~ 5 days, dating from 1992 to present day and is freely available through the NASA PO.DAAC site (http://podaac.jpl.nasa.gov). Here, we used the 1/3 degree horizontal grid spacing. Further details can be obtained directly from the OSCAR webpage (https://www.esr.org/oscar_index.html). The ROMS surface velocities were resampled to fit OSCAR data horizontal resolution and averaged for the same depth and time interval.

## Results

### Seasonal SST fields

The northern part of the domain off Cabo Frio is representative of an important hydrodynamical regime associated to mesoscale activity (e.g., upwelling, surface fronts and eddies). The second profile represents a region where the northbound excursions of a relatively cool-low salinity water tongue are commonly observed along the inner- and mid-shelves [27, 28, 29]. Although still on debate, this northward intrusion is sometimes referred to as the Brazil Counter-Current [30].

**Fig 3:**
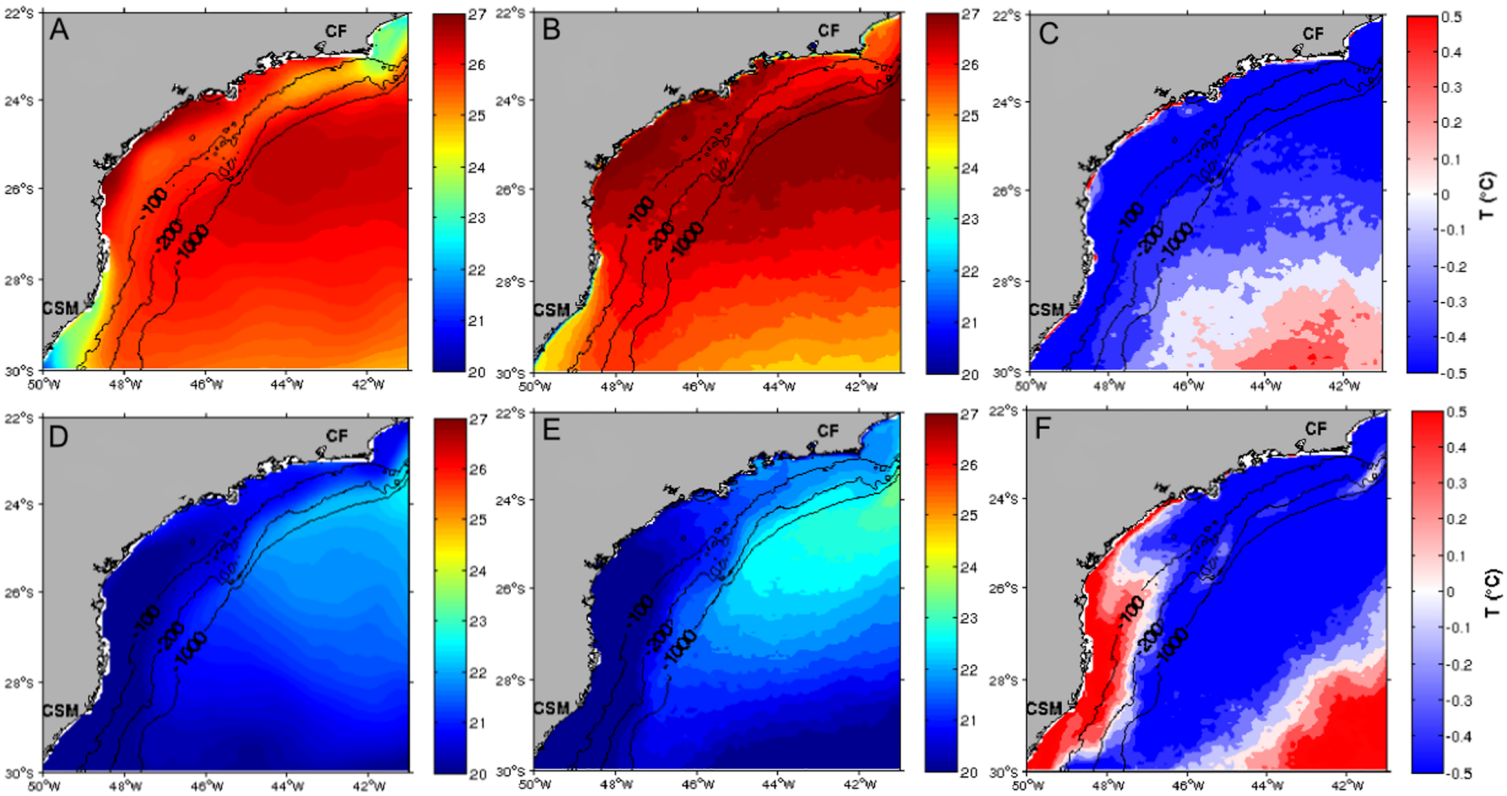
SST ROMS AVHRR. Seasonal SST (°C) means in the SBB for summer (top maps) and winter (bottom maps) resulting from ROMS (A and D), extracted from AVHRR (B and E) data and the differences ROMS minus AVHRR (C and F). CSM, Cabo de Santa Marta and CF, Cabo Frio.

During austral summer (JFM) surface waters over the SBB show temperature values between 25°C and 27°C, while off Cabo Frio and off Cabo de Santa Marta these values are between 22°C and 24°C. Surface temperatures inside the SBB are roughly homogeneous, a reflection of Tropical Waters dominated by the southward flow of the BC warm waters. Although local heating is known to be an important factor, the orientation of the isotherms suggests that an increase in surface temperature is primarily due to the advection of warm waters from lower latitudes [31]. A common onshore-offshore SST gradient is observed at both ends of the domain (Fig 3). The northern one is typically related to the ageosthropic wind-induced upwelling events [32, 33], while the southern one is associated to the northern excursions of cool shelf waters from higher latitudes.

During austral winter (JAS), however, minimum surface temperatures tend to be parallel to the coastline and a strong thermal gradient develops offshore. In the SBB, SST values range from 20°C to 23°C, except for latitudes lower than 24°S, where surface temperatures are 1°C slightly warmer at the most (Fig 3E). South of this latitude, the southern portion of the domain is dominated by the northward intrusion of cold coastal waters reaching up to 25°S and a strong thermal contrast (> 3°C) over the shelf-break can be observed [28, 31]. Average temperatures higher than 23°C extending from shelf to slope regions typically mark the southernmost limit of the Brazil Current. The austral winter SST difference map (Fig 3F) shows temperature values ranging from +0.2°C to +0.5°C south of 26°S for water depths roughly shallower than 100-m, which encompasses the inner and mid-selves in the SBB. These positive values extend farther south of Cabo de Santa Marta.

**Fig 4:**
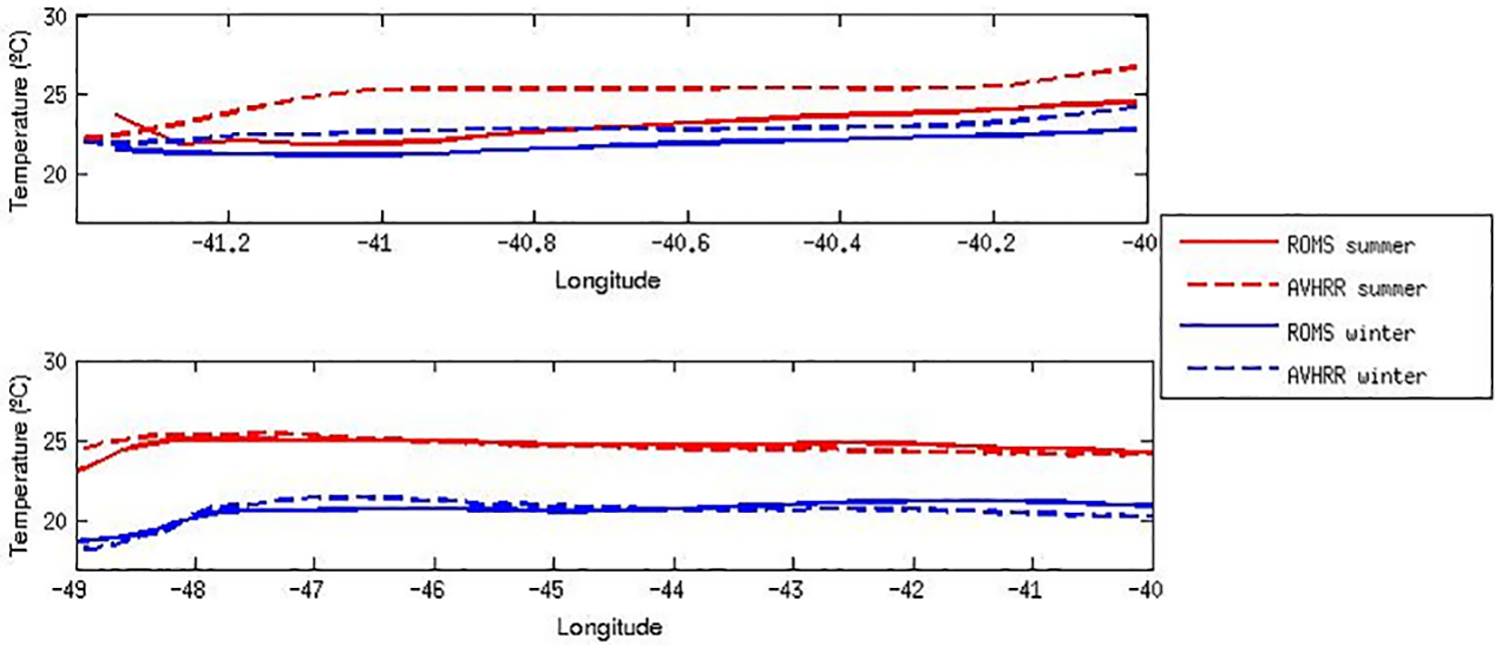
Profiles of SST seasonal means. Horizontal profiles of SST seasonal means (°C) resulting from ROMS and from AVHRR satellite observations, along latitudes 22°S (top panel) and 29°S (bottom panel).

In order to scrutinize these differences, longitudinal profiles of seasonal averaged SST for the northern (22°S) and southern latitudes (29°S), which encompass the SBB region, are shown in Fig 4 respectively. Relatively small-to-large differences are located close to the shore in both northern and southern profiles. For the northern profile, our results show relatively large differences (~ 1.5°C) located close to the shore ~ 41°W during austral summer, and values below 1°C basically for the entire longitudinal extension during austral winter (Fig 4, top panel). On the other hand, smaller temperature differences (> 1°C) are observed for both austral summer and winter southern longitudinal profiles between 49°W and 47°W (Fig 4, bottom panel). Although analogous findings reported by [6] are almost the same as ours, our results are slightly better than theirs in the SBB region.

### Seasonal surface velocities

**Fig 5:**
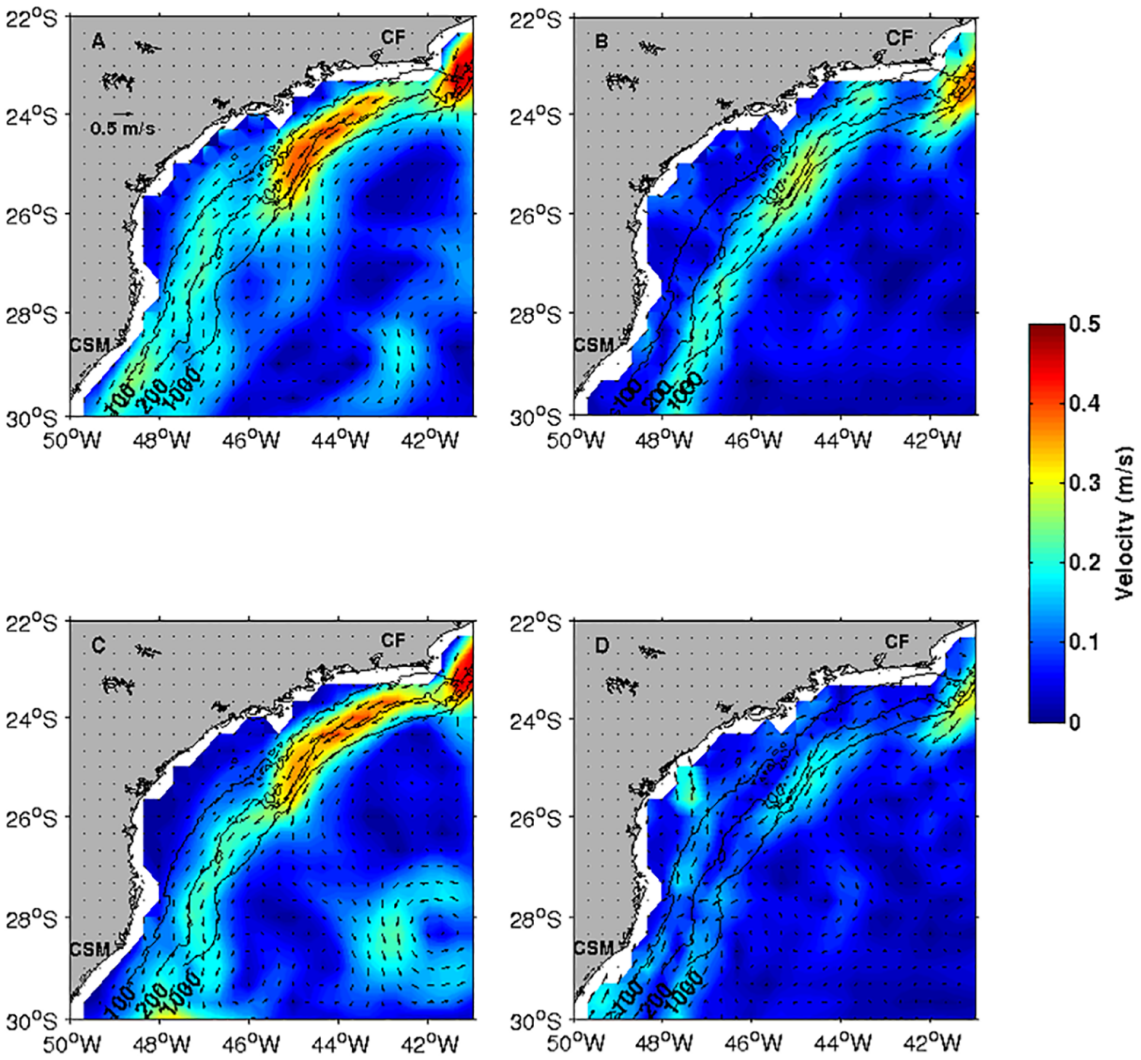
Mean flow ROMS OSCAR. Comparison between summer (top maps) and winter (bottom maps) surface current fields from ROMS outputs (A and C) and OSCAR (B and D). CSM, Cabo de Santa Marta and CF, Cabo Frio.

ROMS and OSCAR seasonal mean surface velocity patterns are consistent and in good agreement, capturing the mesoscale features associated with the Brazil Current meanders and eddies in the SBB. During seasonal austral summer (Fig 5A and 5B), current direction and its associated velocity intensity off Cabo Frio are consistent with observation. The model tends to slightly accelerate the flow over the continental shelf and slope, but the southward flow that dominates the entire SBB domain is well defined in both ROMS and OSCAR data (Fig 5A and 5B). For the seasonal austral winter, a similar pattern is also observed (Fig 5C and 5D), except for the inshore section of the SBB north of Cabo de Santa Marta up to 25°S, where a northward velocities are detected. Indeed, these maps show that our simulations are very close to the expected surface dynamic behavior of the SBB.

### Sardine spawning and larval survival

**Fig 6:**
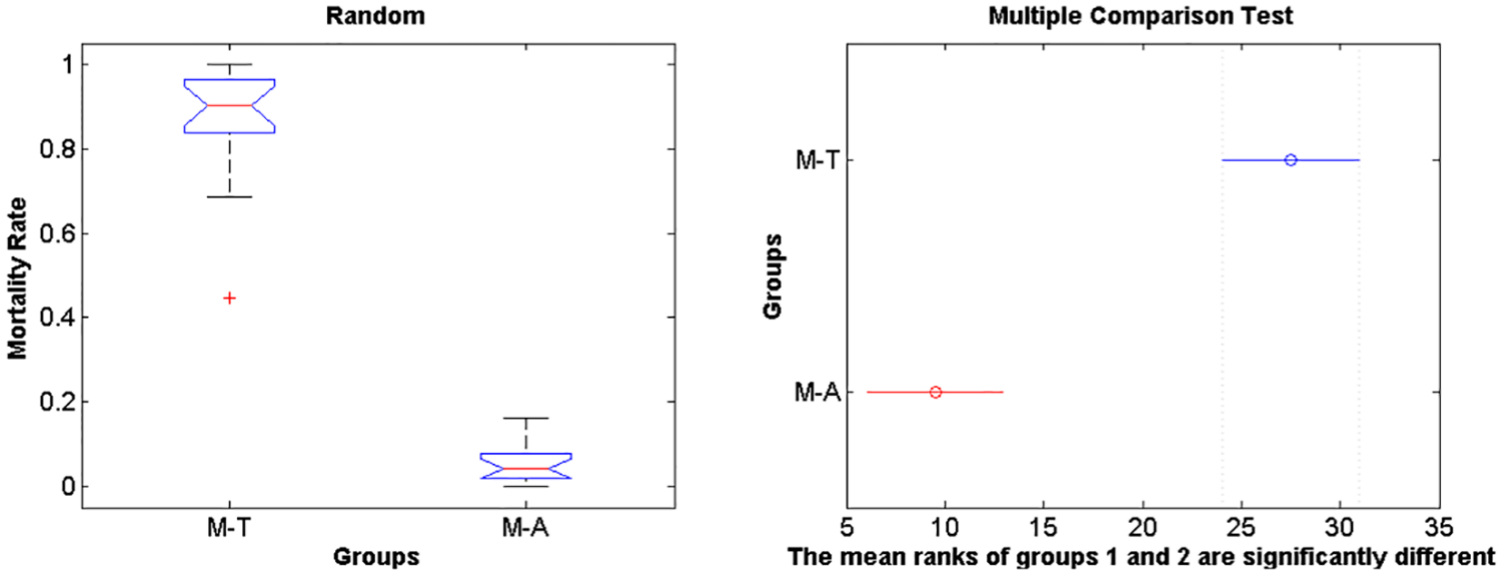
Rank random. Boxplot for differences in mortality rates of larvae by temperature and advection for the random spawning experiment. M-T holds for mortality by temperature and M-A holds for mortality by advection.

**Fig 7:**
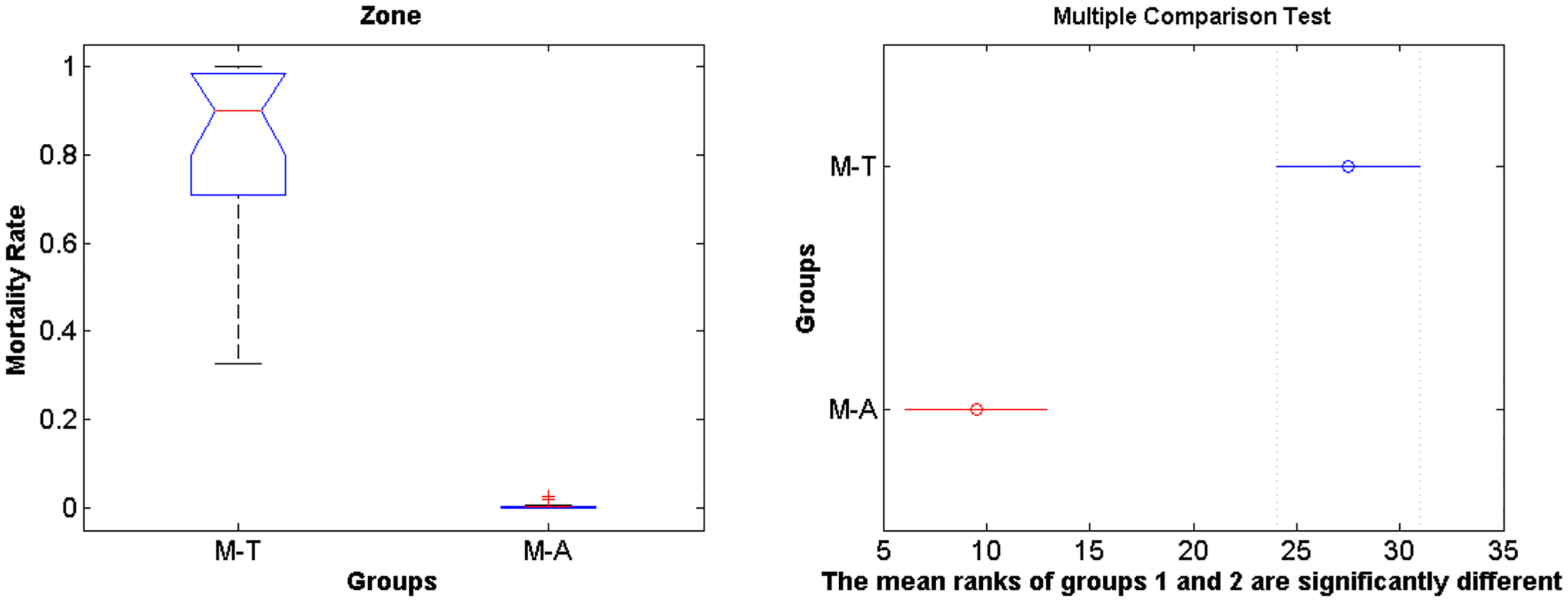
Rank zone. Boxplot for differences in mortality rates of larvae by temperature and advection for the zone spawning experiment.

**Fig 8:**
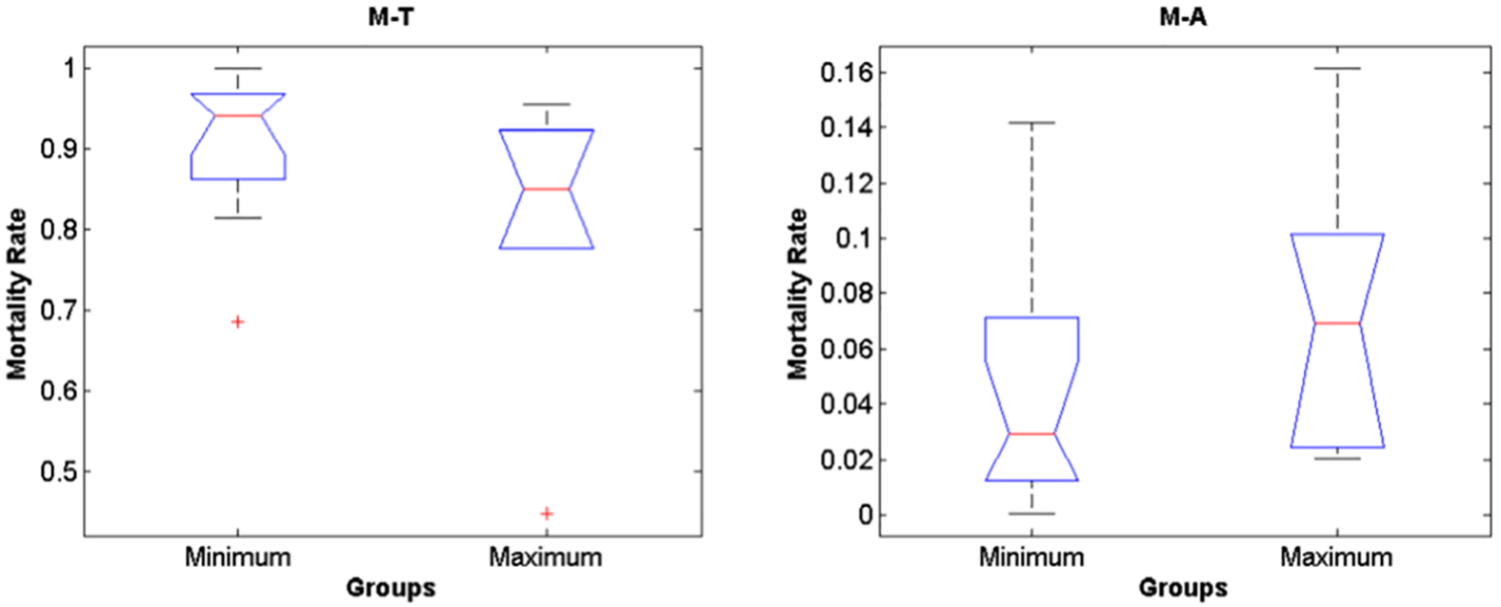
Random maximum and minimum. Boxplot for differences in mortality prior to years of maximum and minimum sardine landings for the random spawning experiment.

**Fig 9:**
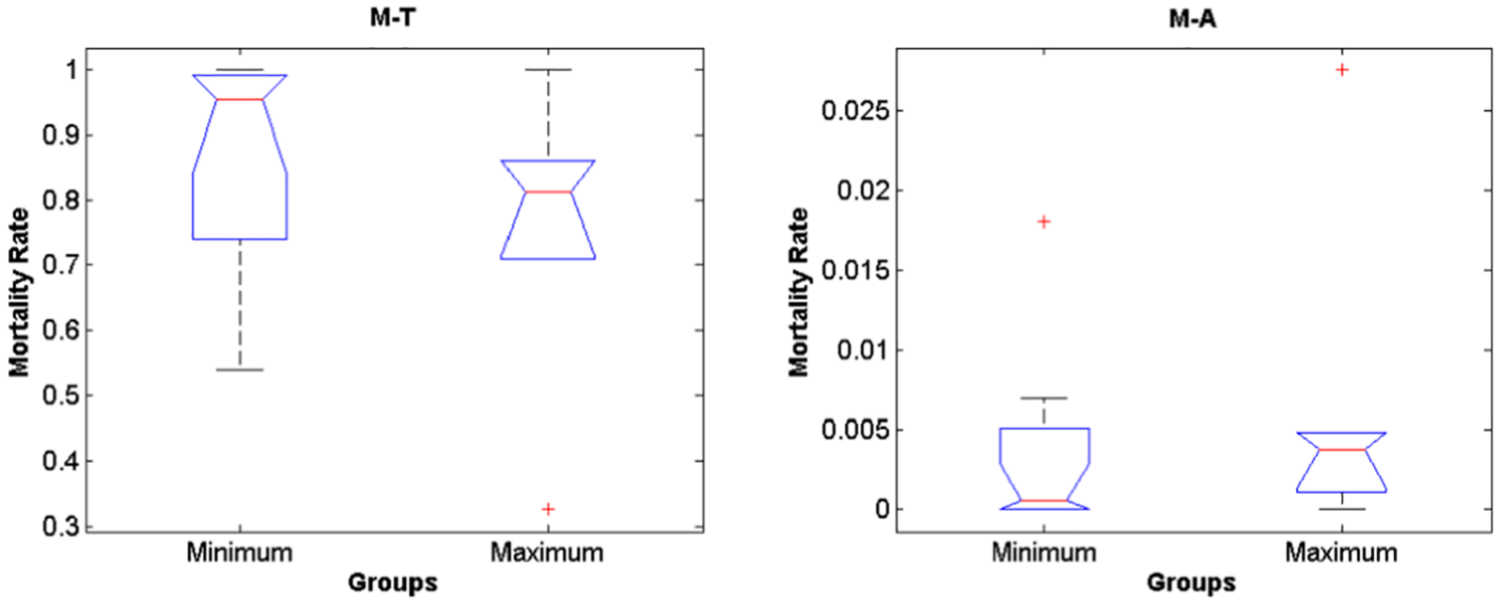
Zone maximum and minimum. Boxplot for differences in mortality prior to years of maximum and minimum sardine landings for the zone spawning experiment.

The IBM simulations showed that mortality of eggs and larvae by low temperature prior to maximum and minimum landings are significantly higher compared to mortality caused by offshore advection (Figs 6 and 7, S1, S2 and S3 Tables, note that rank differences also show they come from different distributions), for both random spawning along the SBB and in spawning zones (*p* = 2.97e-7 and *p* = 2.92e-7, respectively). Overall, around 90% of mortality is explained by low lethal temperature, being higher than mortality by advection in all four spawning zones with variance increasing from south to north for all years. However, no significant differences in mortality (temperature and advection) were detected when maximum and minimum sardine landings are compared (Figs 8 and 9, S1, S2 and S3 Tables). In this case, random and zone spawning do not show significant differences in mortality rates either.

The median of mortality due to low temperature is higher for years prior to minimum landings (though not significantly different), which agrees with the idea that unfavourable SST would negatively impact recruitment and production [34]. The opposite happens for the median of mortality by advection that is lower for extreme low landing years. It is worth noting that the simulations for 1989 and 2000, predating extreme low sardine landings (see Fig 2), showed the highest mortalities of larvae of around 99.5%.

**Fig 10:**
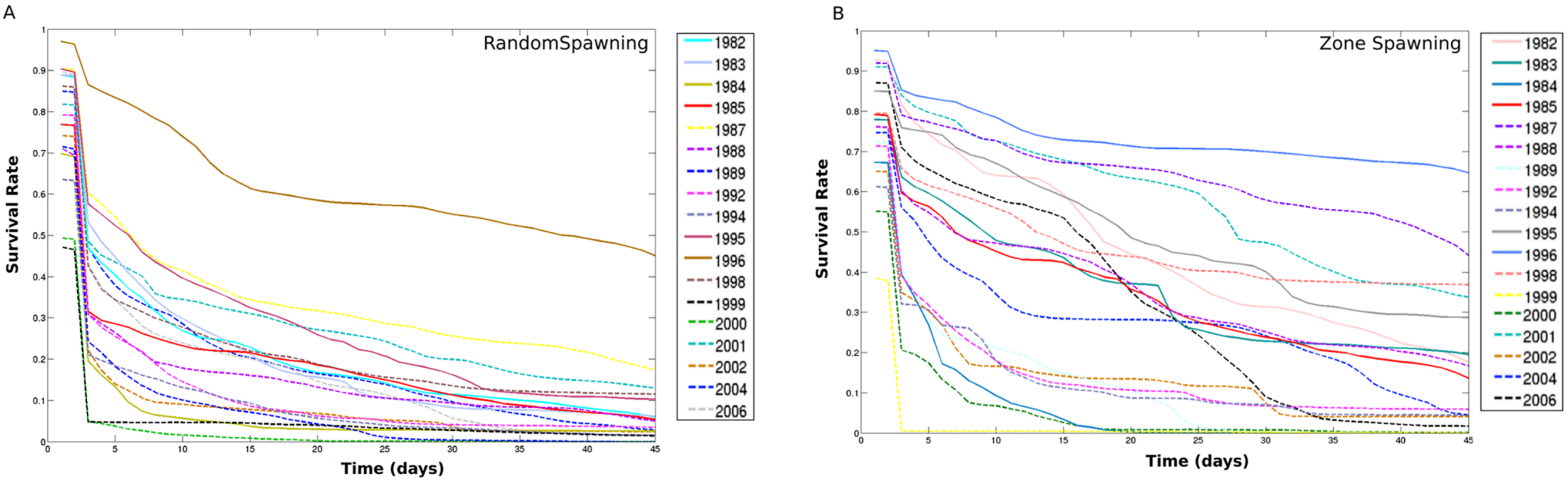
Survival random and zone. Survival rates of eggs and larvae for simulations of random (A) and zone spawning (B). Years of minimum landings are represented by continuous lines and years of maximum landings by dashed (-) lines. Each curve represents a complete simulation (45 days) one year before the statistically selected landing years (see Methods section).

**Fig 11:**
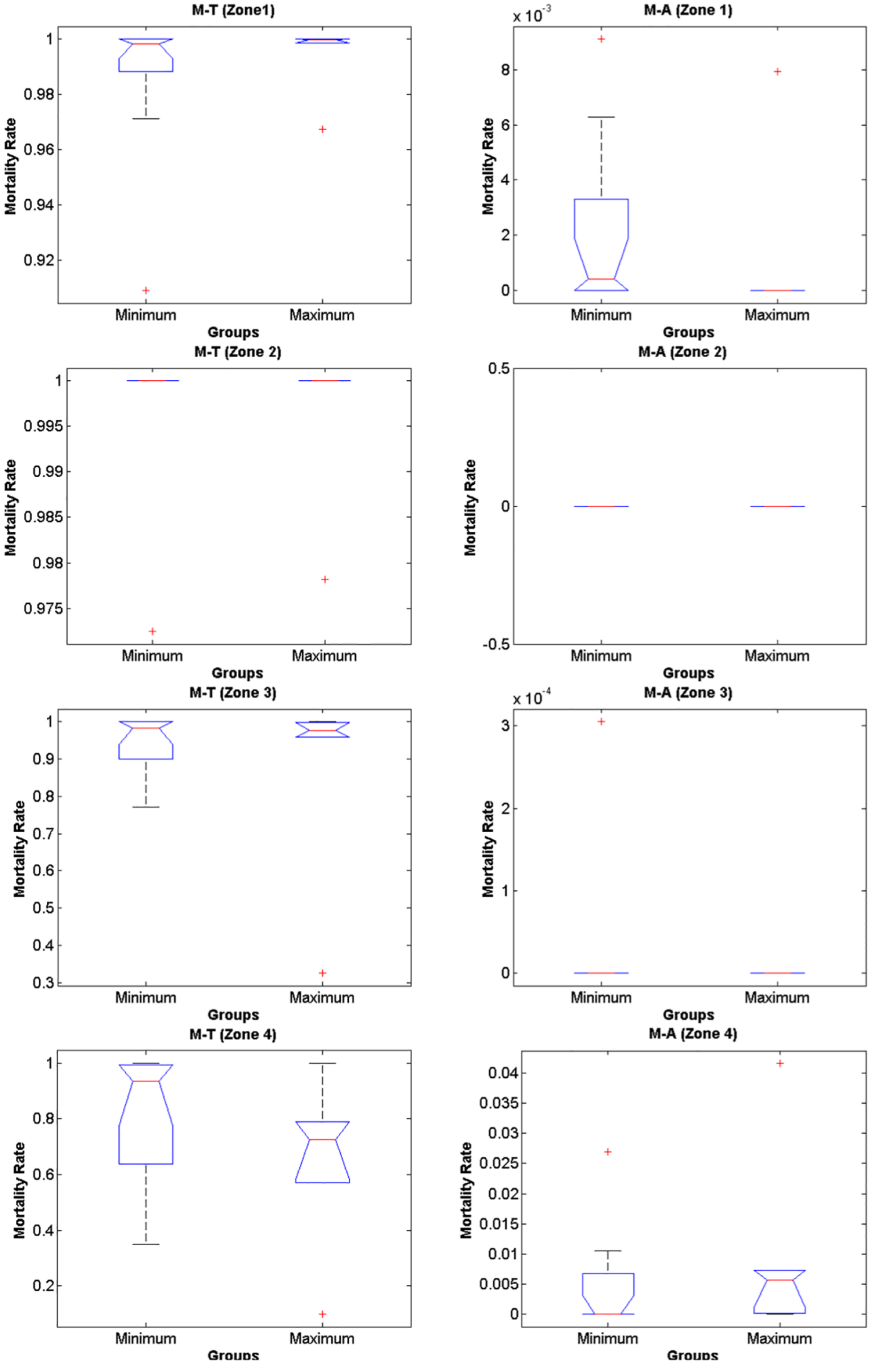
Maximum and minimum Z1-4. Tests for differences in mortality for each of the selected spawning zones between years prior to minimum and maximum landings. Note that there are no significant differences in mortality rates.

Nearly half of the total eggs released in the random spawning experiment die out (considering lethal temperature and advection) in the first five days of simulation, with little differentiation of years of maximum and minimum landings along the time evolution of survival rates (Fig 10A, S1 and S4 Tables). A different behaviour is depicted from the spawning zones experiment, where survival rates slowly decrease along the 45 days of simulations. The years of maximum sardine landings tend to group around higher survival figures (Fig 10B, S1 and S4 Tables). Also, there are no significant differences in mortality rates, between years of maximum and minimum landings, when the spawning zones are analyzed separately. Mortality rates by temperature among years show that it tends to be higher prior to minimum landings and increase from south to north along the SBB (Fig 11, S2 and S3 Tables).

**Fig 12:**
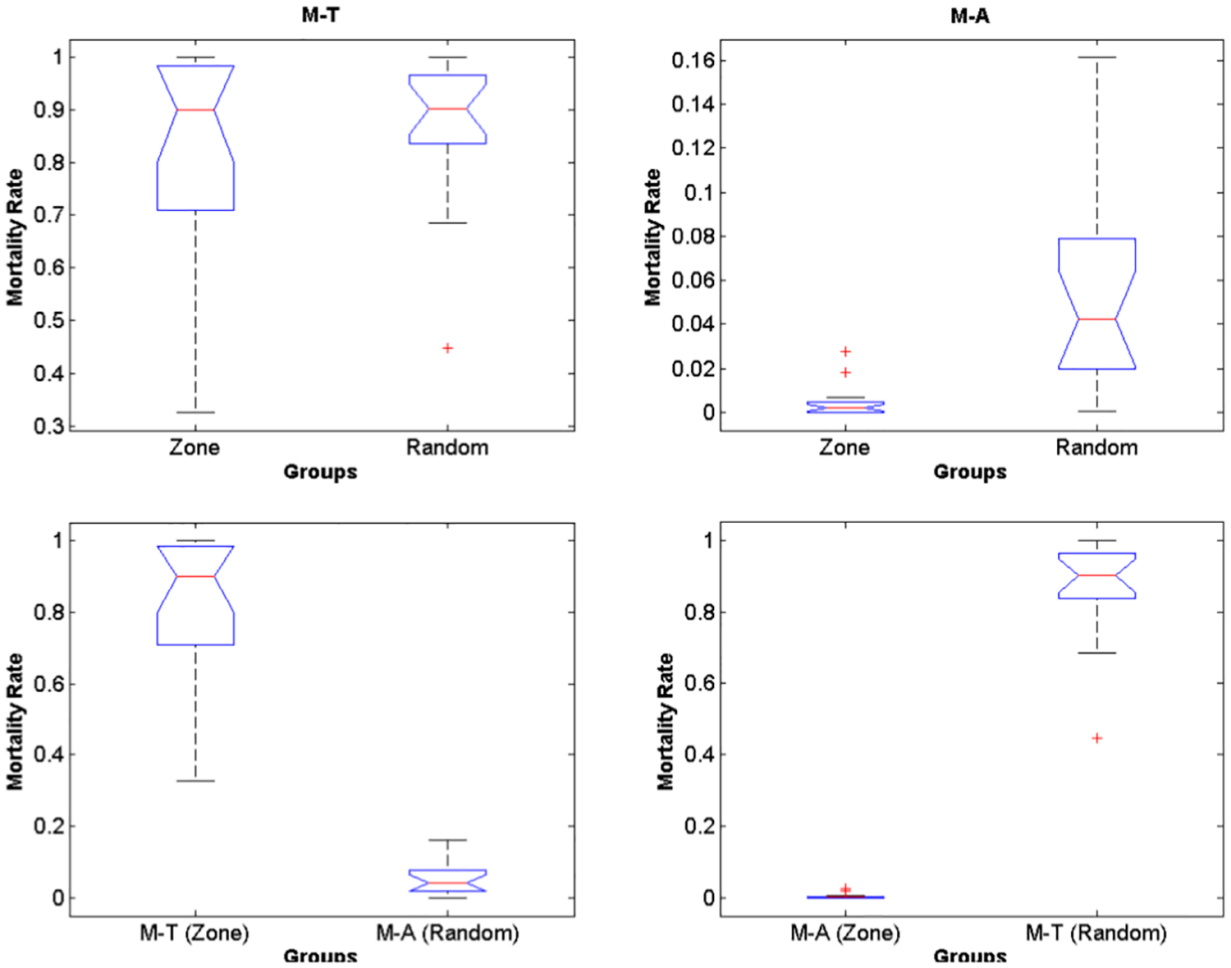
Random zone. Boxplot for random and zone spawning with respect to mortality rates by low lethal temperature and advection. Note that only mortality by advection is significantly higher for random spawning.

**Fig 13:**
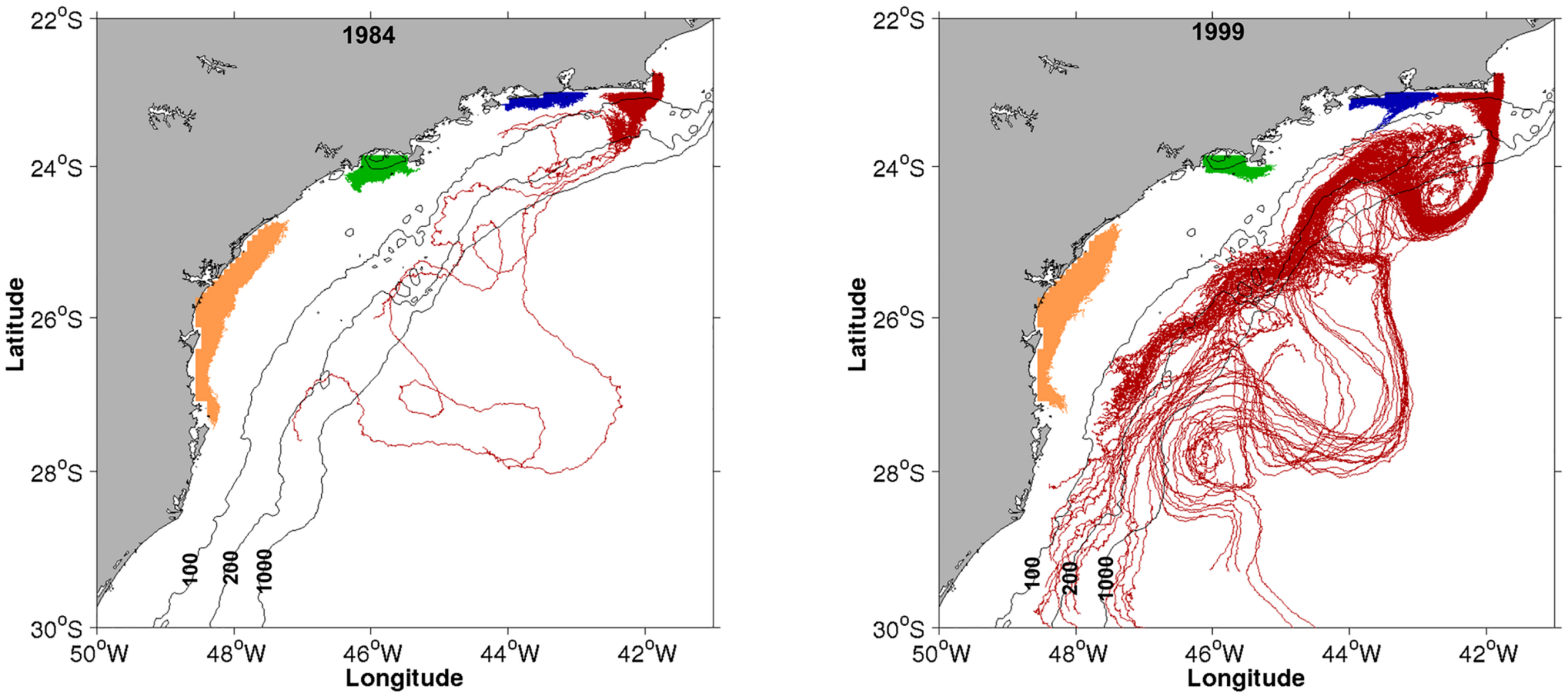
Zone spawning. Examples of total larval trajectories for the zone spawning experiment representing the years of 1984 (left map) and 1999 (right map). Spawning zone Z1, orange; Z2, green; Z3, blue and Z4, red.

**Fig 14:**
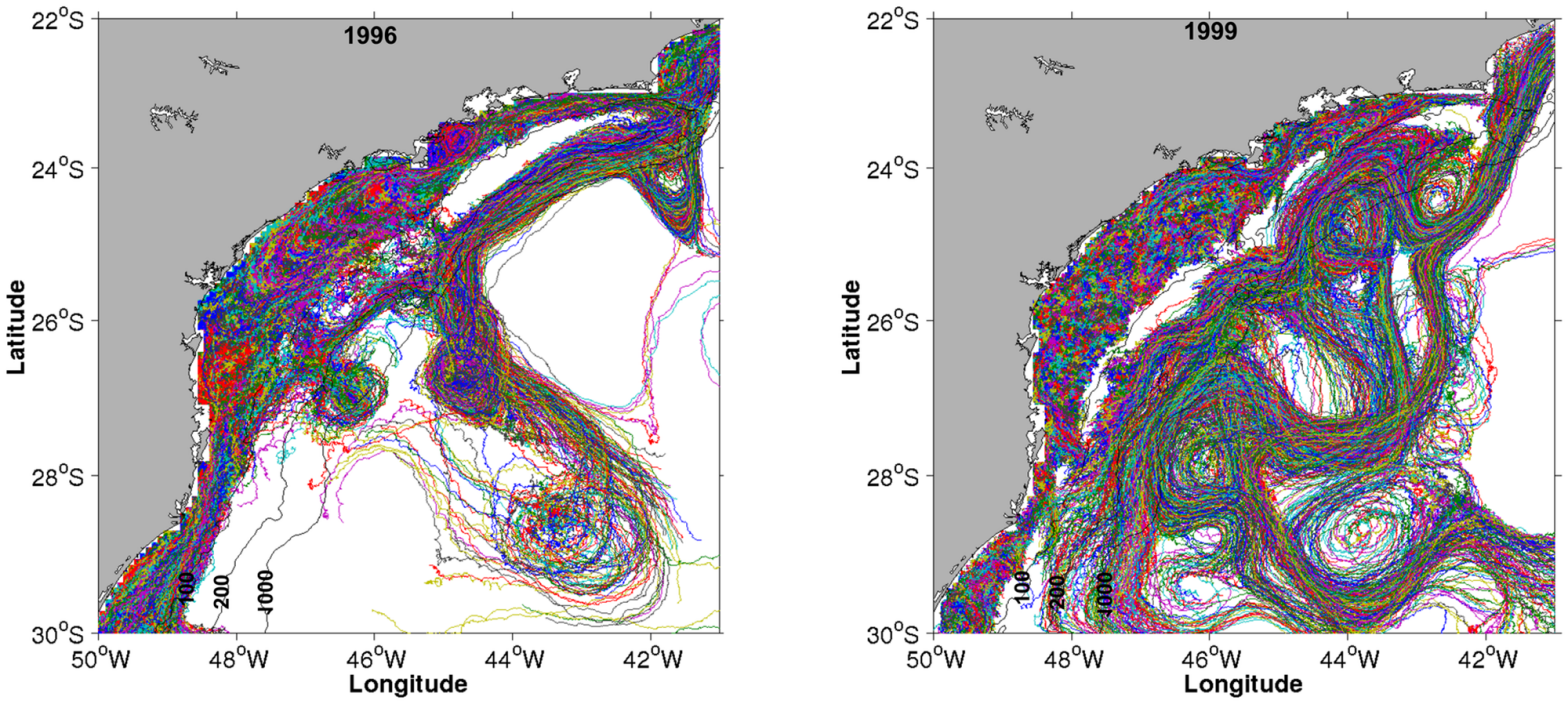
Random spawning. Examples of total larval trajectories for the random spawning experiment representing the years of 1996 (left map) and 1999 (right map). Spawning zone Z1, orange; Z2, green; Z3, blue and Z4, red.

The test for differences between random and zone spawning with respect to mortality rates by low lethal temperature and advection (Fig 12, S1–S3 Tables), showed that only mortality by advection is significantly higher for random spawning (*p* = 1.25e-5, Table 1). Simulated dispersion patterns show that spawning within the predefined zones (Fig 13) favours retention and medium-scale spatial structure of larvae. In contrast, random spawning (Fig 14) increases offshore advection and larvae are more likely to get captured by the BC meanders and eddies because release points include a larger portion of the continental shelf. Therefore, randomly spawned eggs are more susceptible to different (coastal *vs*. offshore) surface circulation regimes within the same spawning season.

**Table 1:** Test Kruskal-Wallis.

**Mortality T Zone—Mortality T Random**					
Source	SS	df	MS	Chi-sq	Prob
Columns	1.78	1	1.778	0.02	0.8993
Error	3883.22	34	114.212		
Total	3885	35			
**Mortality A Zone—Mortality A Random**					
Source	SS	df	MS	Chi-sq	Prob
Columns	2116	1	2216	19.09	1.24852e-5
Error	1764	34	51.882		
Total	3880	35			

Kruskal-Wallis tests for differences in mortality by temperature and advection between the random and zone spawning.

## Discussion

The SST difference map shows a good consistency between ROMS and AVHRR datasets, as the temperature differences range from +0.5°C to -0.5°C for the whole area. Differences in SST during the summer show similar values along the SBB for water depths shallower than 200 m and show that model output is relatively colder than the Pathfinder data, with surface temperature differences ranging from -0.5°C to -0.4°C. Elsewhere, SST differences shift from relatively low negative values to positive values south of 28°S and east of 47°W (see Fig 3C). Average cooler summer SST (compared to satellite data) observed in our model results could possibly increase larval mortality but this -0.5°C bias is spatially homogeneous and is unlikely to seriously affect our interannual analyses. Higher (~ 1.5°C) SST differences found in the northern domain of the model are concentrated close to the shore and could be the result of positive SST bias in the AVHRR products (see below).

Besides the fact that the model accurately reproduces the satellite-derived SST overall, it also resolves the mesoscale dynamical processes quite well, including frontal structures, meanders, eddies along the Brazil Current and the local upwelling region off Cabo Frio. For instance, cooler waters observed in the model outputs in the vicinity of the Cabo Frio are good indicators of modeled and observed upwelled waters and mesoscale cyclonic features previously described in this area [35, 36]. The modeled SST is especially efficient when compared to the AVHRR-derived SST estimates along the coastline, where the infrared retrievals may be cloud-contaminated. The fine mesoscale surface structures evident in the model outputs seem to be smoothed in the infrared SST maps, possibly because the lower spatial resolution of the satellite data. Indeed, [37] argue that this is probably due to the combination of individual images and infrared products to estimate the SST, with differences observed mainly in the austral summer and coastal region around 22°S. Although this is a well-known cloudy area under the influence of the SACZ [38, 39], our results are in better shape than the ones of [6] for the same region and season, and using almost the same model configuration. On the other hand, the modeled SST in the southern portion of the SBB seems to be slightly warmer than the AVHRR data (Fig 3F), a region where the northward penetration of cool and low salinity waters occurs [28, 29]. As pointed out by [40], this northward-propagating coastal water into the SBB is related to the alongshore component of the wind-stress anomaly and freshwater discharge during ENSO events. Therefore, as the model configuration has an open boundary in the south (~ 30°S), which does not include the river discharges of both Rio de la Plata and the Patos Lagoon, it is reasonable to believe the lack of these inputs into the open boundary is responsible for the relatively warmer temperature differences confined south of 26°S and to water depths shallower than 100-m in the southern domain.

The simulations of eggs and larvae mortality caused exclusively by SST and particle advection assume that all other possible causes of mortality are held constant. Also, catch ability has not been considered, which is known to vary with the aggregation pattern, ultimately influencing fish availability and commercial landings over the short term. We have shown that mortality of early life stages is generally very high in the first few days of the random spawning experiment with little variation between years of high and low sardine landings. In nature, the fate of the majority of eggs and larvae is death, not only by temperature stress and advection to unfavourable sites that may lead to starvation, but also by predation or cannibalism. So, the chances of survival are extremely low requiring specific hydrological conditions, and together with density dependent processes, makes it very difficult to predict recruitment and future catches [2].

It has been suggested that the Pacific sardine (*Sardinops sagax*) survival and recruitment are related to environmental conditions during the early life stages and cumulative effects on the adult population before spawning [9]. This cumulative effects could be related to the quality and quantity of body fat, resulted from metabolic advantages obtained from migration of the spawning stock. Our results suggest that the movement of adults to specific spawning sites along the SBB significantly improves larval survival and may impact the performance of sardine fisheries. Despite the lack of statistical relation between mortality by temperature in selected spawning sites and extreme high or low sardine landings, survival of larvae spawned in these sites tends to be higher prior to maximum landings (see Fig 10, S1 and S4 Tables). The point here is that spawning within smaller areas (compared with random spawning) and at specific locations along the SBB improves larval survival but does not impede fish yield to decline, so abrupt fluctuations of fish landings might be related to other factors.

Assuming that recruitment is a product of egg production (very large) times the survival rate (very small), then it is possible that small variations in survival would cause significant changes in the number of recruits, assuming a constant spawning stock. It has been long suggested that the necessity of high food ingestion by low to medium latitude (warm sea) fishes increases the likelyhood of death by starvation [41]. As growth and mortality rates tend to increase with temperature and larval stage durantion trends to decrease, then fast turnover of larval populations is expected. [42] have argued that the interaction of environmental factors and juvenile fish behaviour such as movement and mortality risk associated to movement has a strong impact on juvenile survival and recruitment relationships. So, our results suggest that it is not possible to rely solely on abiotic/extrinsic factors to explain the observed extremes yields of the Brazilian sardine fishery. Other likely influencing factors known to affect the survival of early fish life stages [43] such as predation and starvation should be seriously considered for management purposes.

We know that sardine eggs densities (ind. m^-2^) within the SBB are spatially structured on medium and local scales (50–200 km), while larvae densities are structured on broad- and medium-scales (200–500 km) [7]. The year-to-year spatial structuring of sardine eggs is very complex with the occurrence of all spatial scales (local, medium and broad) in one year, to medium spatial scale structure in another and back to the occurrence of all three scales. The spatial variability of egg densities may also evolve in the short term (few weeks), specially when the spawning activity decreases by the end of summer [7]. The above spatial structures for eggs and larvae could be the result of habitat selection and surface ocean circulation but these are not inevitably reflected in the commercial landings. It is worth noting, however, that broad-scale structuring might be explained by mesoscale processes, coincident with higher interannual variability in mortality rates by temperature north of the SBB (spawning zone 4) prior to periods of minimum landings.

Changes in food abundance, hydrological conditions and the need for spawning have triggered sardine migrations during its evolution, but these are likely to vary on different time scales [44, 45]. In fact, our simulations of particle dispersion show that interannual variability of trajectories in the SBB is extremely high, though it contributes much less to mortality than low SST. Undoubtedly, there are advantages for sardine schools to move around in search for suitable spawning sites so that the strategy of spawning “at the right place and at the right time” [2] could maximize the reproductive success of a few surviving individuals.

The environmental effects during the post-recruitment period were not considered in the present study and they can impact fish mortality, growth rates and sexual maturation [2]. [9] showed that the use of environmental indices such as the Pacific Decadal Oscillation and good adult condition prior to reproduction can significantly improve the prediction of Pacific sardine recruitment. Robust relations linking fish population response to environmental conditions and climate change are, however, difficult to achieve by correlation studies only, as these tend to break down. On the other hand, the IBM approach using high quality physics from an eddy-resolving hydrodynamic model backed by remote sensing and analysis data has allowed the development of end-to-end models. These integrate a number of sub-models that could resolve hydrodynamics, lower and upper trophic levels in one single modelling framework [12, 46, 47]. It is expected that in the near future, ecosystem-based fisheries management initiatives will profit from improved simulations of bottom-up and top-down processes, with the potential to address the global status of fish stocks.

## Conclusions

Despite the limitations imposed by our modelling scheme, namely the lack of some important ecological interaction mechanisms such as predation or cannibalism, our results added a much-needed physical element to our understanding of the Brazilian sardine dynamics. Our modelling scheme realistically simulated increased initial larval mortality caused by temperature, and showed that mortality by advection becomes an important process depending on the spawning strategy. Most importantly though is that oceanic variability alone, particularly during the early developmental stages of the Brazilian sardine, has not proven to be sufficient to explain the recorded extreme fish landings.

The use of a hydrodynamic numerical model to simulate environmental conditions, associated with individually tracked particles mimicking the biophysical behavior of fish eggs and larvae, is critical for a balanced analysis of the spawning process. The spatial structure of sardine eggs and larvae is influenced by the possibility of entraining different hydrodynamic regimes. It is also important to be able to test alternative hypotheses from which one can draw more realistic inferences from different, though possible, spawning scenarios.

## Supporting information

S1 Table Egg and larvae mortalities (number of individuals) for the random spawning experiment. (DOCX)

S2 Table Egg and larvae mortalities (number of individuals) caused by temperature for the zone spawning experiments. (DOCX)

S3 Table Egg and larvae mortalities (number of individuals) caused by advection for the zone spawning experiments. (DOCX)

S4 Table Total mortalities (number of individuals) of egg and larvae for the zone spawning experiments. (DOCX)
