# Supplementary material for: Do the Brazilian sardine commercial landings respond to local ocean circulation?
Source: PLoS One. 2017 May 10;12(5):e0176808. doi: 10.1371/journal.pone.0176808 (PMC5425177; doi:10.1371/journal.pone.0176808)
Supplement: S1 Table — (DOCX) [file pone.0176808.s001.docx]

S1 Table Egg and larvae mortalities (number of individuals) for the random spawning experiment.

| Year | Mortality temperature | Mortality advection | Total mortality |
| --- | --- | --- | --- |
| 2000 | 29980 | 14 | 29994 |
| 2001 | 25683 | 423 | 26106 |
| 2002 | 29134 | 139 | 29273 |
| 2004 | 26027 | 3125 | 29152 |
| 2006 | 28939 | 670 | 29609 |
| 1982 | 25847 | 2363 | 28210 |
| 1983 | 23311 | 4844 | 28155 |
| 1984 | 28641 | 606 | 29247 |
| 1985 | 27660 | 739 | 28399 |
| 1987 | 20544 | 4255 | 24799 |
| 1988 | 26381 | 2122 | 28503 |
| 1989 | 28523 | 1477 | 30000 |
| 1992 | 27888 | 1076 | 28964 |
| 1994 | 28946 | 602 | 29548 |
| 1995 | 25115 | 1792 | 26907 |
| 1996 | 13436 | 3054 | 16490 |
| 1998 | 24389 | 2150 | 26539 |
| 1999 | 29246 | 334 | 29580 |
