# Supplementary material for: Do the Brazilian sardine commercial landings respond to local ocean circulation?
Source: PLoS One. 2017 May 10;12(5):e0176808. doi: 10.1371/journal.pone.0176808 (PMC5425177; doi:10.1371/journal.pone.0176808)
Supplement: S3 Table — (DOCX) [file pone.0176808.s003.docx]

S3 Table Egg and larvae mortalities (number of individuals) caused by advection for the zone spawning experiments.

| Year | Zone 1 | Zone 2 | Zone 3 | Zone 4 |
| --- | --- | --- | --- | --- |
| 2000 | 0 | 0 | 0 | 0 |
| 2001 | 35 | 0 | 0 | 156 |
| 2002 | 0 | 0 | 0 | 0 |
| 2004 | 13 | 0 | 0 | 83 |
| 2006 | 0 | 0 | 1 | 0 |
| 1982 | 0 | 0 | 0 | 146 |
| 1983 | 30 | 0 | 0 | 2 |
| 1984 | 0 | 0 | 0 | 0 |
| 1985 | 0 | 0 | 0 | 125 |
| 1987 | 3 | 0 | 0 | 539 |
| 1988 | 0 | 0 | 0 | 210 |
| 1989 | 11 | 0 | 0 | 0 |
| 1992 | 0 | 0 | 0 | 0 |
| 1994 | 24 | 0 | 0 | 0 |
| 1995 | 0 | 0 | 0 | 102 |
| 1996 | 0 | 0 | 0 | 828 |
| 1998 | 0 | 0 | 0 | 113 |
| 1999 | 6 | 0 | 0 | 0 |
