# Supplementary material for: Do the Brazilian sardine commercial landings respond to local ocean circulation?
Source: PLoS One. 2017 May 10;12(5):e0176808. doi: 10.1371/journal.pone.0176808 (PMC5425177; doi:10.1371/journal.pone.0176808)
Supplement: S4 Table — (DOCX) [file pone.0176808.s004.docx]

S4 Table Total mortalities (number of individuals) of egg and larvae for the zone spawning experiments.

| Year | Zone 1 | Zone 2 | Zone 3 | Zone 4 |
| --- | --- | --- | --- | --- |
| 2000 | 3836 | 2896 | 3330 | 19882 |
| 2001 | 3525 | 2898 | 2979 | 10479 |
| 2002 | 3786 | 2917 | 3269 | 18806 |
| 2004 | 3512 | 2977 | 3354 | 18830 |
| 2006 | 3814 | 2936 | 2947 | 19790 |
| 1982 | 3781 | 2821 | 3067 | 15035 |
| 1983 | 3690 | 2871 | 3308 | 14279 |
| 1984 | 3755 | 2947 | 3343 | 19952 |
| 1985 | 3683 | 2947 | 3332 | 15955 |
| 1987 | 3699 | 2951 | 2543 | 7559 |
| 1988 | 3740 | 2912 | 2963 | 15381 |
| 1989 | 3670 | 2891 | 3341 | 20098 |
| 1992 | 3706 | 2890 | 3296 | 18342 |
| 1994 | 3793 | 2870 | 3253 | 18772 |
| 1995 | 3775 | 2855 | 3259 | 11495 |
| 1996 | 3829 | 2917 | 1073 | 2778 |
| 1998 | 3683 | 2858 | 3038 | 9358 |
| 1999 | 3736 | 2984 | 3292 | 19963 |
